# Variation in male‐built nest volume with nesting‐support quality, colony, and egg production in whiskered terns

**DOI:** 10.1002/ece3.8162

**Published:** 2021-11-04

**Authors:** Jean‐Marc Paillisson, Rémi Chambon

**Affiliations:** ^1^ University of Rennes, CNRS, ECOBIO [(Ecosystèmes, biodiversité, évolution)] ‐ UMR 6553 Rennes France

**Keywords:** *Chlidonias hybrida*, colony size, laying date, nesting‐support stability, postmating sexual signal, reproductive traits

## Abstract

Nest building can represent an energetically costly activity for a variety of animal taxa. Besides, the determinants of within‐species variation in the design of nests, notably with respect to natural and sexual selection, are still insufficiently documented. Based on an observational study, we examined the influence of nesting conditions (nesting‐support quality, colony, laying date, and year) on male‐built nest volume and also its potential role as a postmating sexually selected display in the whiskered tern *Chlidonias hybrida*. This tern species is a monogamous colonial bird with obligate biparental care breeding on aquatic vegetation. Hence, large nesting platforms are expected to be a selective advantage because they would better withstand adverse environmental conditions and provide a secure structure for eggs. Nest size may also serve as a postmating sexual trait, and variation in egg production would be positively associated with nest size. We found that nest volume was adjusted to different environmental cues. A positive relationship was found between nest volume and nesting‐support quality, indicating that the leaf density of white waterlily is essential for nest stability. Variation in nest volume was not correlated to colony size but varied among colonies and years. Male‐built nest volume was also positively associated with mean egg volume per clutch but not with clutch size. The fitness consequences of building a large nest are yet to be studied, and additional investigations are recommended to better understand whether the activity of males early during breeding season (e.g., nest building and courtship feeding performance) really serves as postmating sexually selected signals.

## Graphical abstract

We empirically investigated several hypotheses explaining variation in male‐built nest volume in whiskered terns. Nest volume was mainly associated with nesting conditions (nesting‐support quality, colony, and year) but also with females' egg production (mean egg volume per clutch).

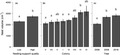

## 1 INTRODUCTION

Nest building is a critical stage in the lifetime of a variety of animal taxa. Nests can exhibit large between‐ and within‐species variation in their design, but their causes and the relative fitness consequences are not still well determined (Hansell, 2000, but see the recent work of Nagy et al. (2019)). The primary function of a nest is to provide a secure structure for eggs during their development and nestlings during rearing (in the case of altricial species). Additional functions include environmental adjustment, crypsis against predators, and parasite control (e.g., Deeming & Mainwaring, 2015; Hansell, 2000). This, in turn, makes it more difficult to determine the, sometimes conflicting, selective pressures responsible for variation in nest characteristics. Nest features (materials, form, structure, size, placement, and duration of nest building) have been indeed shown, mainly in birds, to be influenced by a variety of environmental (extrinsic) factors including the local availability of materials, the microclimatic conditions, the nature and/or quality of the nesting support, the time of year, and the risk of nest predation (Collias & Collias, 1984; Deeming & Mainwaring, 2015; Hoi et al., 1994; Palomino et al., 1998; Persson & Öhrström, 1996). They are also influenced by individual state variables including sex, body size, experience, and quality (Hansell, 2000; Muth & Healy, 2011; Soler et al., 1998).

For species nesting colonially, an increasing body of literature supports the idea that a variety of life‐history (e.g., clutch size, body condition, and/or size) and behavioral (e.g., aggressiveness or, conversely, tolerance toward conspecifics, vigilance) traits and their fitness consequences vary with social environment, notably with group size (i.e., a phenotypic sorting of individuals based on their competitive ability, see the review of Brown (2016) and references therein). Although a large panel of phenotypic traits tend to vary among colonies (but not necessary with colony size; Brown, 2016), variation in the design of nests with colonial nesting remains a neglected issue, whereas nest characteristics may substantially affect various indicators of reproductive success (Deeming & Mainwaring, 2015; Hansell, 2000). More precisely, given that nest‐building behaviors can be energetically costly due to the numerous trips that individuals make to gather the necessary nesting materials (Collias & Collias, 1984; Hansell, 2000; Mainwaring & Hartley, 2013) and that nesting materials can be limited, competition for nesting materials is expected to be exacerbated in large colonies (Carrascal et al., 1995). Furthermore, Moreno et al. (1995) reported that nesting material stealing by conspecifics is particularly associated with colonial nesting. Hence, colony size may influence nest size (Carrascal et al., 1995).

Nest building has also been proposed to have a sexually selected component (e.g., Soler et al., 1998). In birds, the energy costs during breeding are among the highest throughout their lifetime (Williams, 1986), and breeders may therefore trade their effort between current breeding, their own survival, and upcoming breeding events (as predicted by life‐history theory: Gustafsson et al., 1994; Liker & Székely, 2005; Stearns, 1992). In this respect, adults able to assess the quality of their mates as future parents on the basis of sexual signals would experience a critical advantage in adjusting their own reproductive effort (Burley, 1986; Hoelzer, 1989; Møller, 1994; Soler et al., 1998). An extensive body of literature has provided evidence that, in monogamous species with obligate biparental care, the activity of males early during the breeding season (e.g., nest building and courtship feeding performance) may serve as a postmating sexually selected display allowing females to assess the quality of males and to invest differentially in reproduction (reviewed in Collias & Collias, 1984; Hansell, 2000; Wachtmeister, 2001). For instance, Soler et al. (2001) showed that Eurasian magpie (*Pica pica*) females laid larger clutches when nests were experimentally enlarged to simulate increased nest‐building effort by males. A number of studies, notably in passerines, support the idea that females favor large nests (e.g., Moreno et al., 1994; Palomino et al., 1998; Soler et al., 1998).

**FIGURE 1:**
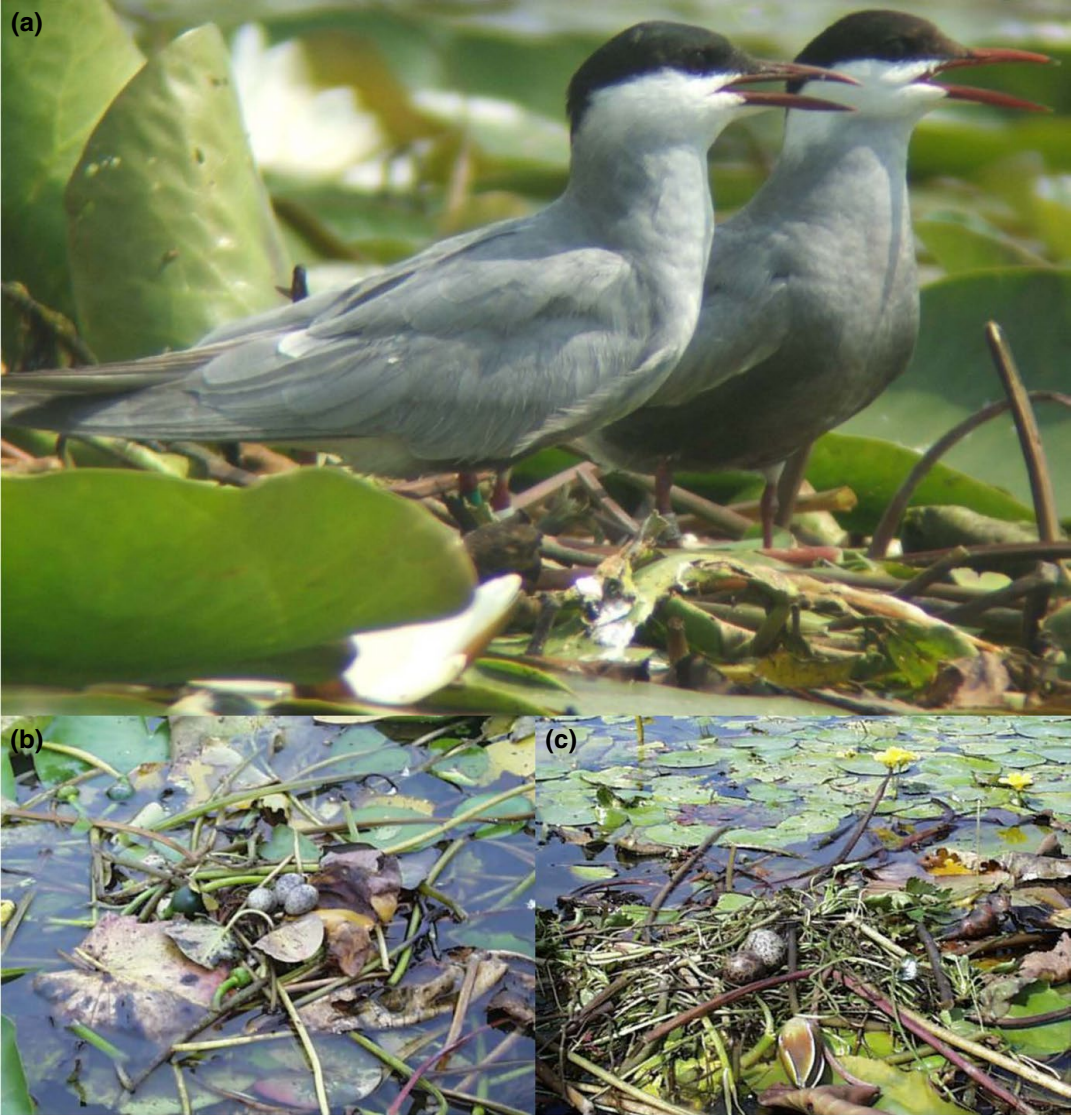
A whiskered tern pair on a dense white waterlily bed (a), and a variety of nests (b, c)

The whiskered tern *Chlidonias hybrida* is a colonial long‐lived migratory monogamous bird with obligate biparental care that, by contrast to most terns (Collias & Collias, 1984; Gochfeld & Burger, 1996), builds floating open‐nesting platforms on aquatic vegetation beds (Bakaria et al., 2002; Paillisson et al., 2006). It makes its nests particularly vulnerable to environmental conditions (floods, and wave and wind actions). Notably, it may be less adaptive to build a nest on sparsely aquatic vegetated beds (e.g., Collias & Collias, 1984). More broadly, previous works support the idea that whiskered terns are sensitive to the quality of nesting support (Paillisson et al., 2006; see a similar conclusion in black terns *Chlidonias niger*; Van der Winden et al., 2004). More exactly, birds settle every year when a minimum waterlily biomass is reached (Paillisson et al., 2006). In addition, we have shown in the past that, during early breeding, whiskered tern females spend the major part of their time at the nesting place, whereas males nearly alone bring nesting plant materials (85% in Chambon et al. (2020)) and also provision their mate (Chambon et al., 2020; Paillisson et al., 2007). Nest building per se is often not very sophisticated since breeders loosely gather plant materials to constitute a floating platform (Figure 1). Therefore, nest size rather results from the accumulation of plant materials brought by whiskered tern males.

The aims of this study are twofold. First, we document the extent of nest size variation in the whiskered tern (Figure 1); this aspect of its breeding biology has been little documented to date (but see Bakaria et al., 2002; Mužinić & Delić, 1997). Second, we consider different nonexclusive hypotheses for nest size variation. The nest support hypothesis posits that nesting materials are used to form a solid base for the nest, increasing its stability (Collias & Collias, 1984). It predicts that nest size should be negatively correlated with the nesting‐support stability (e.g., rock reinforcement under the nest (Collias & Collias, 1984), area of the base of the nest in contact with tree branches (Palomino et al., 1998)) since there is no more need for a large amount of nesting material when nesting‐support stability is high. According to this hypothesis, nests of whiskered terns should be smaller on dense aquatic plant beds than those with less support. However, aquatic plant beds are a highly unstable nest support; hence, a competing prediction would be that sparse vegetation could not support large nests. Therefore, whiskered terns would build large nests only on dense floating plants. By doing so, it may constitute a selective advantage because nests would be better anchored to plants, better withstand wind and wave action and water‐level fluctuations, and, ultimately, provide a more secure structure for eggs (i.e., high‐quality nests). In addition, we examine the optimal breeding time hypothesis (Mainwaring & Hartley, 2008). According to this hypothesis, early breeders are expected to access to better quality nesting places (Ketterson & Nolan, 1983; i.e., dense aquatic vegetation in the whiskered tern). Moreover, early breeders are usually considered as high‐quality individuals (Verhulst & Nisson, 2008) and are supposed to take more time to build nests (see, for instance, Deeming & Mainwaring, 2015). In turn, we expect whiskered terns would build larger nests in such conditions. Furthermore, as the season progresses, breeders have less time to build nests and may settle in less densely vegetated beds (i.e., suboptimal unstable nesting places); hence, we expect that late breeders build smaller nests. We also expect that nest size varies with colony; more specifically, high competition for nesting materials is intended in large colonies and would result in smaller nests (Carrascal et al., 1995). Lastly, we examine the potential benefit of nest size with respect to sexual selection theory. We hypothesize that whether the nest‐building activity of males provides reliable information on their participation in future parental effort that females are able to assess, females would invest differentially in current breeding. Hence, a positive relationship would exist between nest size and egg production. To test these various hypotheses, we conducted a three‐year observational study in a French breeding area supporting several whiskered tern colonies. All studied colonies were characterized regarding their size; colony identity, laying date, and year, together with nest volume, were also used as predictors of females' egg production (see also Paillisson et al., 2007).

## 2 MATERIALS AND METHODS

### 2.1 Study site and fieldwork

The study was conducted at the Lake of Grand‐Lieu, in northwestern France (47°05′N, 1°39′W), which supports one of the major breeding populations of whiskered terns in the country (900–1,460 breeding pairs over the study period, 25%–39% of the national population size). Grand‐Lieu is a very large (ca. 4,000 ha in summer), shallow and eutrophic natural freshwater lake with extensive beds of floating macrophytes (around 700 ha depending on the year), including mainly white waterlily *Nymphaea alba* beds where whiskered terns settle in well‐spaced colonies (see Paillisson et al. (2008) for more details). Whiskered terns build nesting platforms from macrophyte fragments, mainly waterlily leaves and stems, and sometimes from common club‐rush *Scirpus lacustris* stems (depending on the proximity of colonies to this nesting material source). The almost circular platforms are generally completed before egg laying. Nest cup lining (when cup is present) is sometimes adjusted by both members of a pair when incubating eggs using soft plant materials (water chestnut *Trapa natans*, yellow‐floating heart *Nymphoides peltata*, and primrose yellow *Ludwigia grandiflora*). In any case, the amount of nesting material used to complete nest cups is very limited compared with that for nesting platforms.

JMP visited all the active nests of eight colonies by boat in 2008, 2009, and 2018 only once during egg incubation to limit disturbance (see colony size in Table 1). The measurement of platform size and egg size takes less than five minutes per nest. Field observations showed that parents came back to the nest within 1–15 min then after. The largest external diameter of the nests was measured using a giant caliper (*d*, to the nearest 1 cm), and their maximum height above the waterline using a bracket equipped with a spirit level (*h*, to the nearest 0.1 cm). Nest size was approximated using the formula of the volume of a cone: (*d*/2)² × *π* × *h*/3 (cm^3^). As previously mentioned, the amount of plant material for nest cups, when present, is very limited compared with that for nesting platforms; hence, we focused on platform size that best indicates the nest‐building activity of males. The dominant plants used for nest building were recorded. A small number of platforms were made mostly of club‐rush (<5% of the total number of nests once all selection criteria are applied); they were discarded from the dataset because they were larger than the nests composed of waterlily (data not shown) and were too few to represent a competing factor in subsequent analyses. The quality of the nesting support was visually estimated by determining the leaf density of white waterlily in a 1‐m radius around nests (i.e., a proxy for its biomass; see Paillisson & Marion, 2006 for more details). Two classes were defined: low (when one leaf layer covers the water surface totally or not) or high (when several floating and aerial leaf layers cover the total water surface).

**TABLE 1:** Colony size and summary of the number of nests (*n* = 297) among the levels of factors used for exploring variation in nest volume and egg production (only colonies I, IV, V, and VI for this latter case; *n* = 225)

Colony	Colony size (nests)			Number of nests to study nest volume and egg production										
	Year			Year			Waterlily leaf density		Laying date		Clutch size			Total
	2008	2009	2018	2008	2009	2018	Low	High	Peak	Late	1	2	3	
I	57	–	19	13	–	13	18	8	7	19	6	13	7	26
II	–	–	18	–	–	12	12	0	12	0	2	4	6	12
III	12	–	–	6	–	–	0	6	6	0	1	4	1	6
IV	22	38	43	12	15	16	22	21	6	37	11	15	17	43
V	–	91	–	–	61	–	54	7	60	1	11	24	26	61
VI	84	61	88	19	37	39	42	53	78	17	15	33	47	95
VII	61	23	–	14	11	–	25	0	25	0	3	10	12	25
VIII	18	46	–	7	22	–	17	12	14	15	5	12	12	29
Total	178	291	168	71	146	80	190	107	208	89	54	115	128	297

Note

Colonies (I, II, III, IV, V, VI, VII, and VIII) represent well‐delimited areas where whiskered terns settle sometimes for several years; otherwise, a dash (–) is used. Year: 2008, 2009, and 2018. Waterlily leaf density (as a proxy of nesting‐support quality): low or high. Laying date: residual clutch initiation date classified as peak or late laying (too few data for the early‐laying class). Clutch size: one, two, or three eggs.

Clutch size and egg size are classically used to describe females’ reproductive traits (Birchard & Deeming, 2015; Brulez et al., 2015). As whiskered terns typically lay up to three eggs (Paillisson et al., 2007), clutches with more than three eggs were discarded (<1% of the total) because they could result from conspecific brood parasitism (Paillisson et al., 2008) and, hence, could not reliably represent the reproductive effort by the host females. Egg volume (cm^3^) was calculated based on the measurement of egg length and width (using a Vernier caliper, to the nearest 0.01 mm) using the equation provided for the kittiwake *Rissa tridactyla* in Coulson (1963): egg volume = 0.4866 × egg length × egg width². Egg weight (measured using an electronic scale to the nearest 0.1 g) was used to estimate egg age using the linear egg density/age relationship we published elsewhere (Paillisson et al., 2007). Egg age was also used to determine whether one‐ and two‐egg clutches were complete, knowing that an interval of at least 1 day is necessary between the laying of successive eggs. Only clutches defined as complete with certainty were kept for subsequent analyses (see Paillisson et al., 2007). Egg age was also used to estimate the clutch initiation date (i.e., the laying date of the first egg of a clutch). We took the clutch initiation date into account in the analyses because clutch size and/or egg size may vary as the season progresses (due to, e.g., changes in food availability (Parsons, 1975; Sydeman et al., 1991) or replacement clutches (Coulson & Thomas, 1985; Wendeln, 1997)). To do this, we converted the estimated clutch initiation dates (i.e., Julian dates) into residual laying dates (i.e., relative dates) to control between‐year differences in the egg‐laying period. More exactly, the estimated clutch initiation dates were expressed in pentades (5‐day time intervals, beginning of May 20) and converted into residual laying dates by subtracting each clutch‐laying date (in pentades) from the peak laying date of all nests of each year (pentades 5, 7, and 7 in 2008, 2009, and 2018, respectively). Finally, laying dates were classified into three classes: early (pentades ranging from −3 to −2, 4% of the total number of nests used for analysis), peak (pentades −1 to 0, 67% of all nests), and late (pentades +1 to +3, 29% of all nests). The number of nests assigned to the early‐laying class was too small to be retained for subsequent statistical analyses (see sample sizes for the two retained laying classes for each colony in Table 1). Lastly, given that nesting platforms are composed of slowly decaying plant fragments, we assumed that we reasonably evaluated males’ nest‐building effort by keeping only nests with ≤10‐day‐old eggs (i.e., nests whose size was measured during the first half of the egg incubation stage). Overall, the dataset used for analysis included 297 nests, for 4–6 colonies per year (see details in Table 1). In addition, behavioral observations of focal nests collected in 2008 are shown in Appendix 1 to provide an extensive overview of the activity of males before egg laying with respect to sexual selection aspects (see also the Section 4).

### 2.2 Statistical analyses

First, we used linear models (LMs, with a Gaussian error distribution and an identity link function) to assess the relationship between nest volume of whiskered terns (log‐transformed to better fit the model) and the aforementioned variables: nesting‐support quality (e.g., Collias & Collias, 1984), egg‐laying period (peak or late laying; see also Britt & Deeming, 2011; Mainwaring & Hartley, 2008), colony size (Carrascal et al., 1995), colony identity, and year (i.e., the local conditions experienced by adults during breeding; Britt & Deeming, 2011). These analyses were performed on the 297 nests. Second, we examined whether egg production varied according to nest volume (more precisely residuals from the previous model to control for the effects of the aforementioned variables), nesting‐support quality, year, colony size, colony identity, and egg‐laying period (i.e., a putative proxy for breeders' quality: Verhulst et al., 1995; Verhulst & Nilsson, 2008; Wendeln, 1997). As aforementioned, we considered nest volume as a proxy for nest‐building activity of whiskered tern males since they substantially contribute to material delivery during early breeding (85% of the total number nesting material deliveries in Chambon et al. (2020), 97% in Appendix 1). Although the participation of males in nesting plant delivery likely varies between pairs, we were not able to control for this in the analyses since nest‐building behaviors presented in Appendix 1 and nest volume were not collected for the same pairs. We used multinomial logistic regression models (with a multinomial error distribution and a generalized logit link function) to investigate clutch size variation. We examined the relationship between clutch size (the probability of a whiskered tern laying a one‐, two‐, or three‐egg clutch) as the response variable and the candidate explanatory variables. Additionally, we used LMs to examine variation in mean egg volume per clutch as a function of the candidate variables. Clutch size was considered as a candidate predictor in these latter analyses. Due to small or unbalanced sample size issues, the statistical analyses on egg production were performed on a subset of four colonies (*n* = 225 nests; see Table 1).

For all analyses, we produced all candidate models (i.e., testing all possible simple and additive effects, and the null model). Since being correlated, colony size and colony identity were not used together in models. Interaction effects were not tested as no biological hypothesis seemed prevalent. Models were ranked by the Akaike information criterion corrected for small sample sizes (AICc; Burnham & Anderson, 2003). Additionally, *R*² (McFadden *pseudo‐R*² for multinomial models) values were calculated for each model. Models with an additional noninformative variable (i.e., zero included in the 95% confidence interval of its parameter estimate or no enough variation explained to justify its inclusion in the model) within ΔAICc < 2 were discarded to eliminate misinterpretation (Arnold, 2010). Since results were qualitatively similar over other model selections (ΔAICc < 4, ΔAICc < 7, or cumulated weight of AICc (ꙍAICc) < 0.95; see all ranked models in Appendix 2), we presented only the results from ΔAICc < 2. The results of pairwise post hoc comparisons (considering the Tukey‐adjusted *p*‐values) of the adjusted estimates of the response variables (marginal means ± SE derived from the best models) were provided.

All statistical analyses were performed with R 3.5.2 (R Development Core Team, 2018) using the *AICcmodavg*, *car*, *emmeans*, *lme4*, *MuMIn*, *nnet*, *performance*, *r2glmm*, and *RVAideMemoire* libraries. The significance level was fixed at *α* = 0.05.

## 3 RESULTS

**FIGURE 2:**
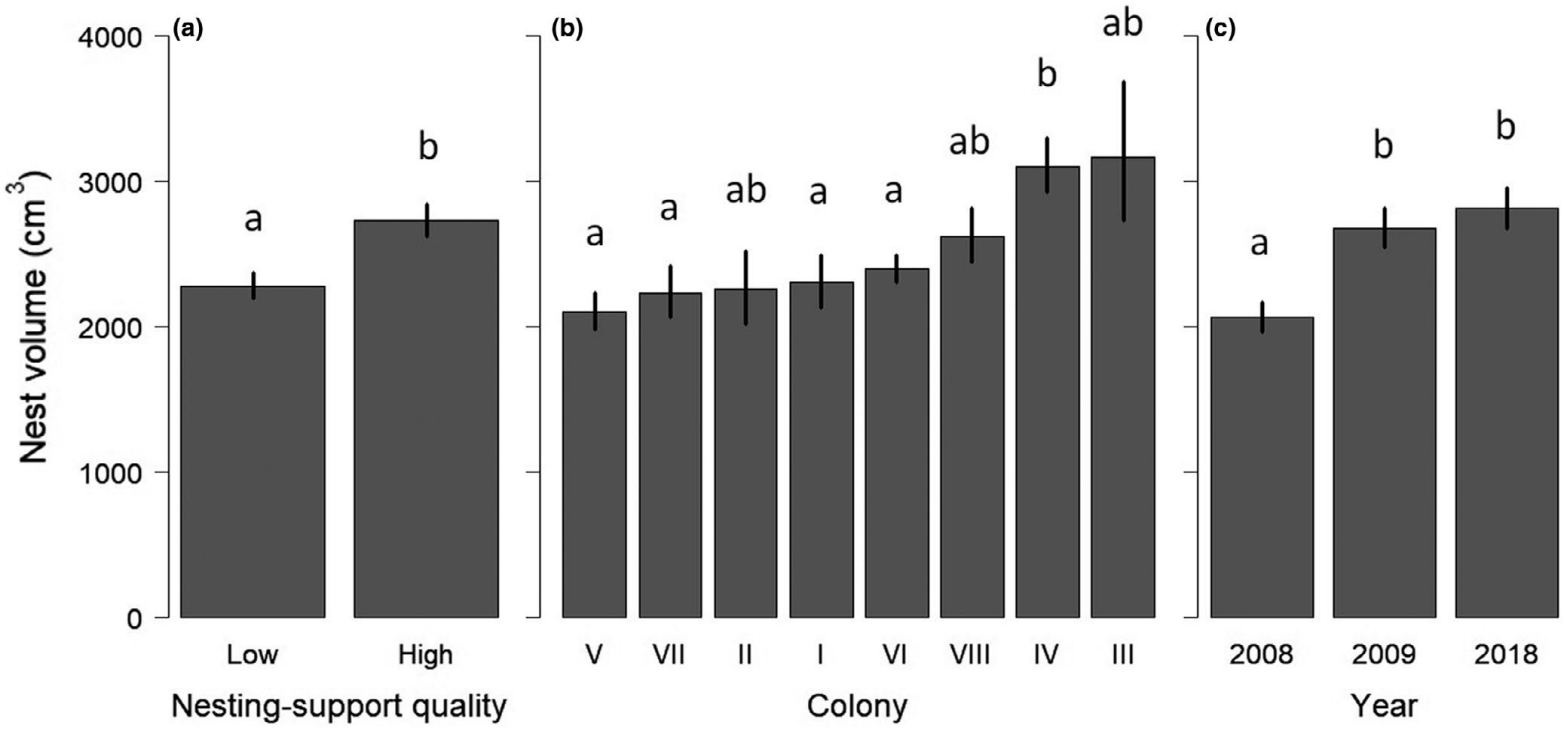
Nest volume (mean ± *SE*, in cm^3^, log) according to: (a) nesting‐support quality (classified according to leaf density), (b) colony identity, and (c) year. Different letters above bars indicate significant differences between factor levels

Estimated raw nest volumes ranged from 477 to 8,701 cm^3^, with a mean value of 2,618 ± 63 *SE* cm^3^. Among the 24 models computed (Appendix 2), the retained model explaining variation in nest volume included nesting‐support quality, colony identity, and year (*R* ^2^ = 0.22; Table 2). Nest volume was on average smaller on sparse than dense aquatic vegetation (2,276 ± 91 and 2,724 ± 109 cm^3^, respectively; Figure 2a). Nest volume was statistically larger in colony IV (3,103 ± 186 cm^3^), intermediate in colonies II, III, and VIII (2,252 ± 248, 3,165 ± 477, and 2,618 ± 183 cm^3^, respectively), and the smallest in all the other colonies (from 2,101 ± 126 to 2,392 ± 96 cm^3^; Figure 2b). There was no significant correlation between nest volume and colony size. Lastly, nest volume was lower in 2008 (2,059 ± 103 cm^3^) than in 2009 and 2018 (2,670 ± 134 and 2,807 ± 140 cm^3^, respectively; Figure 2c).

**TABLE 2:** Model selection with ΔAICc < 2 used to explain variation in nest volume (log) and female's reproductive traits (the probability of a whiskered tern laying a one‐, two‐, or three‐egg clutch, and mean egg volume per clutch)

Response variable	Explanatory variables	(Intercept)	Nesting‐support quality	Colony identity							Year		Egg‐laying period	Nest volume	AICc	ΔAICc	ꙍAICc	R²
			High	II	III	IV	V	VI	VII	VIII	2009	2018	Late					
Nest volume	Nesting‐support quality + Colony identity + Year + Laying period	7.60	0.18	−0.10	0.18	0.34	−0.15	−0.02	−0.13	0.12	0.19	0.25	−0.15	–	256.30	0.00	0.62	0.22
		(0.21)	(0.10)	(0.27)	(0.36)	(0.18)	(0.21)	(0.18)	(0.24)	(0.21)	(0.15)	(0.15)	(0.15)					
	**Nesting‐support quality** + **Colony identity** + **Year**	**7.46**	**0.18**	−**0.02**	**0.31**	**0.30**	−**0.09**	**0.04**	−**0.03**	**0.12**	**0.27**	**0.31**		–	**257.99**	**1.69**	**0.27**	**0.22**
		**(16)**	**(0.10)**	**(0.26)**	**(0.34)**	**(0.18)**	**(0.20)**	**(0.16)**	**(0.21)**	**(0.22)**	**(0.13)**	**(0.14)**						
Clutch size (2‐ vs. 1‐egg)	**Nest volume**	**0.69**												**0.18**	**472.66**	**0.00**	**0.52**	**0.01**
		**(0.37)**												**(1.08)**				
(3‐ vs. 1‐egg)		**0.81**												**1.16**				
		**(0.37)**												**(1.10)**				
(2‐ vs. 1‐egg)	Nest volume + Nesting‐support quality	0.65	0.12											0.18	473.37	0.71	0.22	0.02
		(0.45)	(0.78)											(1.08)				
(3‐ vs. 1‐egg)		0.58	0.60											1.18				
		(0.46)	(0.76)											(1.10)				
Mean egg volume	**Nest volume** + **Colony identity**	**14.39**				**1.00**	**0.58**	**0.80**						**0.43**	**657.03**	**0.00**	**0.15**	**0.09**
		**(0.40)**				**(0.50)**	**(0.47)**	**(0.45)**						**(0.40)**				
	Nest volume + Colony identity + Nesting‐support quality	14.46	−0.20			1.04	0.54	0.86						0.44	657.29	0.26	0.13	0.10
		(0.40)	(0.30)			(0.50)	(0.48)	(0.45)						(0.39)				
	Nest volume + Colony identity + Laying period	14.59				1.03	0.39	0.66					−0.27	0.41	657.43	0.40	0.12	0.10
		(0.49)				(0.51)	(0.55)	(0.50)					(0.40)	(0.40)				
	Nest volume + Colony identity + Nesting‐support quality + Laying period	14.62	−0.18			1.06	0.38	0.72					−0.23	0.41	658.13	1.09	0.09	0.10
		(0.73)	(0.30)			(0.51)	(0.56)	(0.51)					(0.41)	(0.40)				
	Nest volume + Colony identity + Year	14.24				1.01	0.65	0.80			0.08	0.30		0.44	658.60	1.57	0.07	0.10
		(0.45)				(0.52)	(0.59)	(0.47)			(0.44)	(0.40)		(0.40)				

Note

For categorical predictors, estimates (with 95% confidence intervals in brackets) from the retained models (in bold) are provided for each level (the difference in estimated values to a reference level, i.e., colony I, 2008, low leaf density and late clutches for colony, year, nesting‐support quality, and laying period, respectively).

Year: 2008, 2009, and 2018. Colony identity: I to VIII, but only four colonies (I, IV, V, and VI) were considered when females' reproductive traits were the response variables (see the text). Nesting‐support quality: low and high (based on waterlily leaf density). Egg‐laying date: residual clutch initiation date classified as peak or late laying. Nest volume (when used as an explanatory variable) was controlled for the effects of nesting‐support quality, colony, and year (see the text for more details). A dash (–) indicates that the variable was not tested for the given response variable.

**FIGURE 3:**
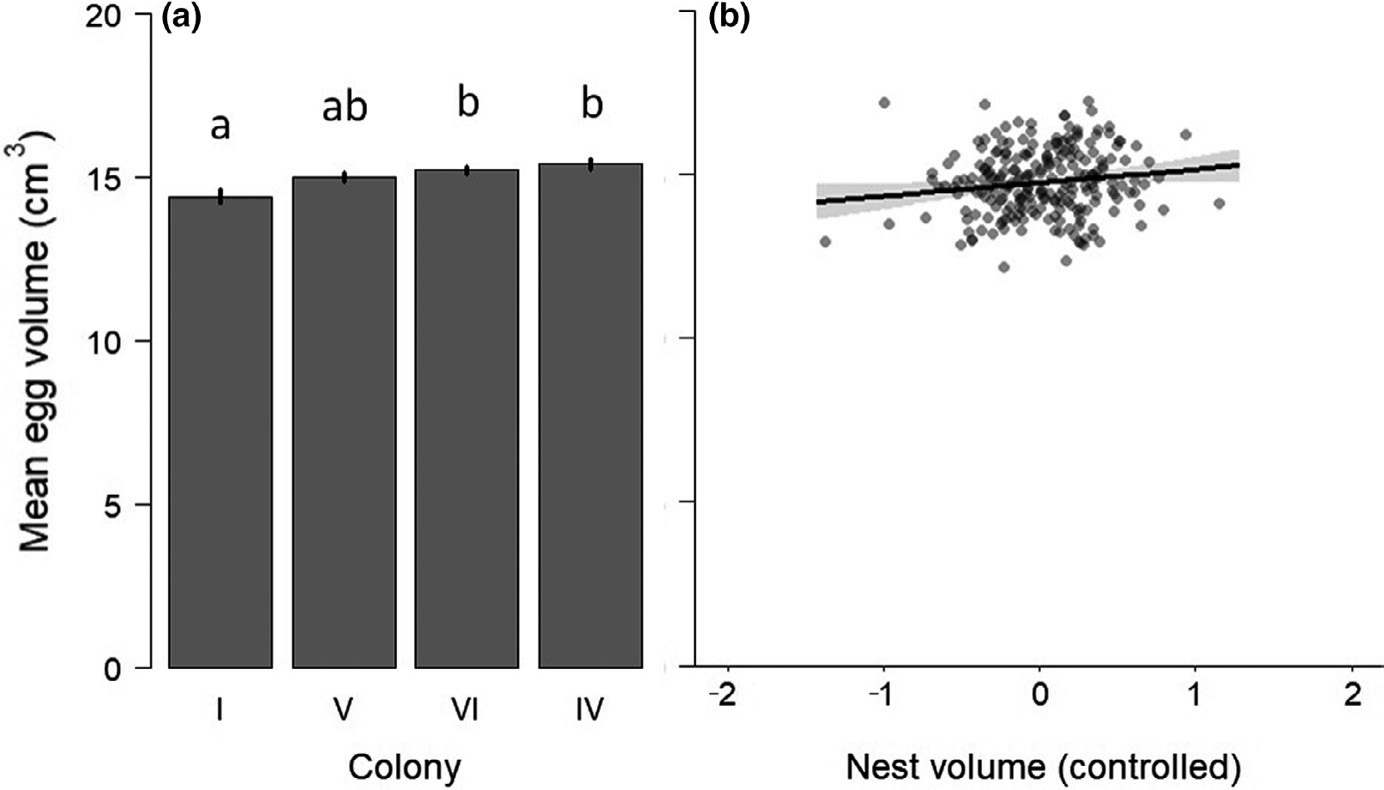
Mean egg volume per clutch (in cm^3^) according to: (a) colony identity and (b) nest volume (after controlling for the effect of the predictors given in Figure 2). Different letters above bars indicate significant differences between colonies, and, the 95% credible interval is provided for the effect of nest volume

One‐egg clutches were much less represented (18%) than two‐egg (39%) and three‐egg (43%) clutches across the complete dataset (*n* = 297 nests). Values were very similar in the subset of four colonies (colonies I, IV, V, and VI) used to explore the effects of candidate variables on egg production: 19%, 38% and 43%, respectively. The retained model explaining clutch size (*n* = 48 models tested; Appendix 2) only included nest volume as predictor (Table 2). However, the *pseudo‐R*² value was very small, indicating that none of the variables used explained variation in the proportion of one‐, two‐, or three‐egg clutches. Concerning mean egg volume per clutch, the retained model (*n* = 96 models tested; Appendix 2) included colony and nest volume as significant predictors (*R*² = 0.09; Table 2). Mean egg volume was the highest in colonies IV and VI (15.39 ± 0.16 and 15.20 ± 0.11 cm^3^, respectively), the lowest in colony I (14.39 ± 0.20 cm^3^), and intermediate in colony V (14.97 ± 0.13 cm^3^; Figure 3a). In addition, mean egg volume slightly increased with nest volume (Table 2 and Figure 3b).

## 4 DISCUSSION

Nest‐building behavior is generally viewed as a result of different selection pressures, and several hypotheses have been suggested to explain nest size variation (e.g., Deeming & Mainwaring, 2015; Hansell, 2000). In the present study, we tested some of these hypotheses in the whiskered tern, which can build nests of largely varying size. We showed that nest volume was adjusted to different environmental cues: nesting‐support quality (i.e., a proxy for nest stability), colony, and possible between‐year variation in the conditions experienced by breeders. We also found that females' reproductive traits (precisely mean egg volume per clutch) were positively correlated to nest volume, which means that nest building could also be a postmating sexual signal, as discussed later.

Contrary to the nesting‐support hypothesis, nest volume was not negatively correlated to the leaf density of white waterlily. Conversely, nests were larger on dense aquatic vegetation and, in turn, this would support the competing prediction we put forward (i.e., sparse vegetation could not support large nests). Moreover, large nests take time and energy to build (Hansell, 2000; Mainwaring & Hartley, 2013; Soler et al., 1998). This is a critical issue notably for migrant species because of the optimal breeding time constraint (Mainwaring & Hartley, 2008); only early breeders (also supposed to be high‐quality individuals; Verhulst & Nilsson, 2008) are expected to have a long nest‐building period and build large nests (see Mainwaring and Hartley (2008), and Smith et al. (2013) in passerines). However, our results are not consistent with this prediction since nest volume was not correlated with laying period. This is somewhat surprising, because late breeders are expected to access to suboptimal nesting places and build smaller nests. Since waterlily biomass increases as the season progresses (Paillisson & Marion, 2011), maybe the quality of nesting places used by later breeders is not reduced. This speculation is quite plausible since late breeders even preferred high‐quality nesting places (53% of the total number of nests were in dense vegetation) compared with early breeders (31%). All this suggest that nesting‐support quality is essential for breeders to build large nests. Large nests likely constitute a selective advantage for whiskered terns because they are better anchored to floating plants. As a result, they are more stable and less sensitive to wind and wave action and water‐level fluctuations. Indeed, dislocated and/or flooded platforms are commonly observed, especially in colonies on sparsely aquatic vegetated beds following a severe storm (JMP, pers. obs.). Future investigations would be, however, required to explore this specific issue. Besides, nest predation pressure, which is a conflicting evolutionary pressure to which nest size can be subject (see, e.g., Mainwaring et al. (2015) and references therein), is likely low in our study site. Magpies *P. pica* and black kites *Milvus migrans* are occasionally observed, and when an individual is detected close to a colony, whiskered terns alarm and massively attack it. Therefore, building large nests remains an advantage for whiskered terns at Lake Grand‐Lieu.

Nest volume also varied between whiskered tern colonies, but not according to colony size. This means that in colonies with high challenging social conditions (i.e., a high number of breeders), competition for nesting material did not result in plant material depletion. One possible explanation is that colonies are not enough densely populated to impact nest volume. Colony size at Lake Grand‐Lieu is indeed moderate compared with elsewhere (Minias et al., 2014). Moreover, large colonies may settle in better habitats (i.e., with enough material for breeders) resulting in nests of similar size compared with less dense colonies. The fact that nest volume varied according to colony identity, irrespective of breeder numbers, rather suggests that environmental conditions differ between colonies (nesting‐support quality, nesting material availability, etc.).

**FIGURE A1:**
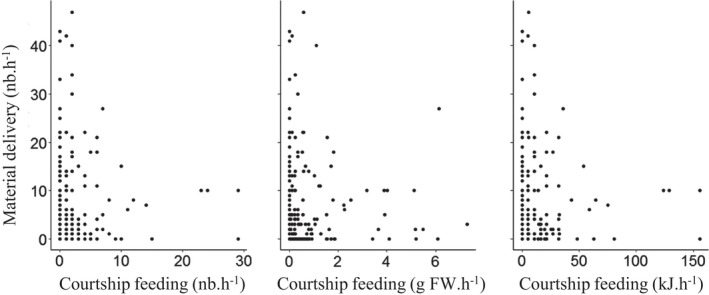
Material delivery rate (number per hour) as a function of courtship feeding by males expressed in (a) abundance (number of prey per hour), (b) biomass (grams of fresh weight per hour), and (c) energy (kilojoules per hour) during nest building (*n* = 205 observation bouts for 44 males). Fish represented 59.6% of the 512 prey captured (277 cyprinids and 28 pike), insects represented 6.4% (28 individuals of *Zygoptera* spp. and 5 other insects), and 174 prey remained undetermined. Fish size ranged from 20 to 110 mm, most of them were <30 mm in length

To function as a sexually selected male trait, nest may primarily reflect a significant participation of males to build it. As aforementioned, time–activity budgets showed that whiskered tern males indeed made practically alone all trips to gather nesting materials (Table A1). Time–activity budgets also revealed that males can highly provision their mate before egg laying. The functions of courtship feeding have long been debated in the past, and several hypotheses have been supported by empirical data including the postmating sexual signal hypothesis (Wachtmeister, 2001, and notably Nisbet (1973) and Wiggins and Morris (1986) in another tern species, the common tern *Sterna hirundo*). Hence, two displays (nesting material delivery and courtship feeding) would potentially act simultaneously as sexual signals in whiskered terns (see also Yoon et al. (2015) in oriental storks *Ciconia boyciana*). We found that males exhibiting the most intensive courtship feeding brought nesting material at a low rate (no negative relationship was, however, observed; Figure A1). Therefore, the information gathered by a female from the activity of its mate during early breeding is highly complex. Moreover, males' effort may vary in the course of the nest‐building period, so that the three‐day period during which their activities were recorded may not have coincided with the period of maximum energy requirements of females for all studied breeding pairs (peak energy requirements occurring, for instance, only 1–2 days before laying in common terns; Moore et al., 2000). Additional investigations are needed to clarify the relationship between nesting material and food supply throughout the whole prelaying period and also to provide a better understanding of the functions of courtship feeding in whiskered terns.

Besides, it is admitted that in order to be a reliable postmating sexual signal, a parent's display has to convey its ability to provide parental care to its partner; the partner, in turn, invest differentially in reproduction to acquire direct fitness benefits. This means that data on chick provisioning by males are needed to examine whether males that invest more in nest building (i.e., build larger nests) also invest more in chick provisioning or not. Unfortunately, we do not possess such data. This requires intensive fieldwork (trapping, banding, and sexing adults) because it is frequently impossible to visually differentiate sexes during intense chick provisioning (but see Ledwoń & Neubauer, 2017). Additional investigations are needed to examine whether the activities of males before egg laying well inform on their future investment in chick provisioning. Nevertheless, variation in mean egg volume per clutch was explained by nest volume. Hence, it cannot be excluded that nest volume could be a sexual signal. Again, additional data are needed to support this hypothesis. Egg volume was also colony‐dependent, without any straightforward explanation; no apparent relationship indeed occurred with the number of active nests. Other factors, notably the own phenotypic quality of females, have been suggested to explain differences in females’ reproductive traits (Coulson & Porter, 1985; Hipfner et al., 1999; Slagsvold & Lifjeld, 1990), sometimes depending on colonies (Brown, 2016). Unfortunately, such data are not available in the present study.

In conclusion, this study yields knowledge on reproductive traits in a poorly studied tern species. More broadly, it also contributes to the identification of drivers of within‐species variation in nest size in birds. Nest‐building behaviors by whiskered tern males were mainly influenced by nesting conditions (nesting‐support quality and colony). Since whiskered terns build large nests when nesting conditions appear suitable, they likely take advantage of this increased costly activity. Hence, the next step would be to assess the relations between nest size and fitness issues, notably breeding success. In addition, nest size possibly functions as a sexually selected male trait in whiskered terns (egg volume was correlated to nest volume); future investigations are needed to explore this issue, also considering courtship feeding.

## CONFLICT OF INTEREST

None declared.

## AUTHOR CONTRIBUTIONS

**Jean‐Marc Paillisson:** Conceptualization (lead); Methodology (lead); Project administration (lead); Writing‐review & editing (equal). **Rémi Chambon:** Formal analysis (lead); Writing‐original draft (equal).

## Data Availability

Data supporting the results (nest volume, egg production, and potential predictors) are supplied as supplementary materials on the Dryad Digital Repository (https://doi.org/10.5061/dryad.n02v6wwz2).

## APPENDIX 1 Nesting material delivery and courtship feeding in whiskered terns

In a previous study, we showed the importance of the intensity of courtship feeding on egg production in whiskered terns at the population level (Paillisson et al., 2007). Therefore, females may use multiple signals from their mate during early breeding, notably nesting material delivery and courtship feeding, to possibly adjust their reproductive effort accordingly. In addition to the study of the selective pressures influencing nest volume (a proxy for males' nest‐building activity; see the main text), we explored whether the males that brought high amounts of nesting material were also those that highly provisioned their mate or not. By doing so, we provided an extensive overview of the activity of males before egg laying that is thought to be useful to interpret variation in females' reproductive effort.

Nesting material delivery and food provisioning to females were recorded for 44 breeding pairs randomly selected in seven colonies, for four to six 1‐hr observation bouts per pair over three consecutive days during nest building (which takes <2 weeks) in 2008 (from 29 May to 11 June, *n* = 205 observation bouts). Observations were made from a boat with a telescope (50–100 m from the nests) during the daytime (7.00–19.00), in nice sunny weather. We differentiated sexes visually (male bills being larger than female bills, Latraube, 2006; Ledwoń, 2011) to gather additional information on the relative contribution of males to nest material delivery compared with already‐published works (Chambon et al., 2020; Paillisson et al., 2007). Typical sex‐specific behaviors (courtship feeding and copulation) help in determining the sex of breeders when bill size differences between individuals are sometimes slight. Typically, birds arrive to the platform with nesting material or prey in the bill. Nest material delivery and food provisioning to females were recorded and expressed in rate (number h^−1^) for each individual. The fieldworker (JMP) was able to visually determine a large part of prey delivered to females (66% of all prey, *n* = 512); they were categorized into invertebrates (mainly *Zygoptera* spp.) or small fish (bream (*Abramis brama*), rudd (*Scardinius erythrophtalmus*), sunbleak (*Leucaspius delineates*), or juvenile pike (*Esox Lucius*)). Fish size was estimated with reference to the length of the bird's bill (to the nearest 5 mm). Courtship feeding was also converted into biomass (grams of fresh weight per hour, g FW h^−1^) and in energy supply (kilojoules per hour, kJ h^−1^), as the energy requirements of females are likely maximum at this time of the breeding season (see Neuman et al. (1998) for black‐legged kittiwakes and Moore et al. (2000) for common terns). Fish biomass was calculated using length/fresh mass equations defined in situ (Adam & Elie, 1993). For *Zygoptera* spp., we used an average biomass value of 0.0365 g per individual calculated from 26 specimens of the most common species present at Grand‐Lieu. For the undetermined prey (i.e., too small prey to be identified), we used the lowest estimated biomass value of fish captured by whiskered terns because fish were the dominant prey (see below). Prey were converted into energy values as detailed in Ledwoń and Neubauer (2017), assuming a food assimilation rate of 0.77 and the following energy values for each prey type: 0.07, 5.38, and 14.34 kJ for *Zygoptera*, fish ≤50 or >50 mm in length, respectively.

Overall, males brought 97% of the 1,397 nesting material recorded (50%–100% of nest material deliveries depending on the breeding pair). Nevertheless, high differences in mean material delivery rate were found between males (0.2–25.5 plant fragments h^−1^). Similarly, mean courtship feeding largely varied between males whatever the measurement unit: 0–12 prey h^−1^, 0–3.7 g FW h^−1^ or 0–64.6 kJ h^−1^. Males displaying the highest material delivery rates were not those providing high food supply (*p* = 0.27, 0.34 and 0.25 for feeding rate, biomass and energy supply, respectively, *R* ^2^ = 0.01, generalized linear mixed models with a negative binomial error distribution, a log‐link function, and the identity of the nests as a random effect, Figure A1).

## APPENDIX 2 Models to explain variation in nest volume and female's reproductive traits in whiskered terns

**TABLE A1:** Models ranked by AICc with associated basic statistics for nest volume, the probability of a female laying a one‐, two‐, or three‐egg clutch, and mean egg volume per clutch

Response variable	Explanatory variables	AICc	ΔAICc	ꙍAICc	*R* ^2^
Nest volume	Nesting‐support quality + Colony identity + Year + Laying period	256.30	0.00	0.62	0.22
	Nesting‐support quality + Colony identity + Year	257.99	1.69	0.27	0.22
	Year + Colony size + Nesting‐support quality	260.54	4.24	0.07	0.18
	Year + Colony size + Nesting‐support quality + Laying period	262.56	6.26	0.03	0.18
	Colony identity + Nesting‐support quality + Laying period	264.34	8.04	0.01	0.19
	Colony identity + Year + Laying period	266.07	9.77	0.00	0.19
	Colony identity + Year	268.45	12.14	0.00	0.18
	Colony identity + Laying period	270.81	14.51	0.00	0.17
	Year + Nesting‐support quality	273.52	17.22	0.00	0.14
	Year + Nesting‐support quality + Laying period	273.96	17.66	0.00	0.14
	Colony identity + Nesting‐support quality	279.15	22.85	0.00	0.15
	Colony identity	283.98	27.68	0.00	0.13
	Colony size + Nesting‐support quality + Laying period	286.49	30.19	0.00	0.10
	Colony size + Nesting‐support quality	286.87	30.57	0.00	0.09
	Year + Colony size	291.55	35.25	0.00	0.08
	Year + Colony size + Laying period	293.05	36.74	0.00	0.08
	Nesting‐support quality	293.30	37.00	0.00	0.03
	Nesting‐support quality + Laying period	295.14	38.84	0.00	0.06
	Year + Laying period	297.70	41.39	0.00	0.06
	Year	297.77	41.47	0.00	0.06
	Colony size	307.13	50.83	0.00	0.19
	Colony size + Laying period	308.84	52.54	0.00	0.02
	*null*	310.94	54.64	0.00	0.00
	Laying period	312.93	56.63	0.00	0.00
Clutch size	Nest volume	472.66	0.00	0.31	0.01
	Nest volume + Nesting‐support quality	473.37	0.71	0.22	0.02
	*null*	475.09	2.43	0.09	0.00
	Nesting‐support quality	475.80	3.14	0.06	0.01
	Colony size + Nest volume	475.85	3.19	0.06	0.02
	Laying period + Nest volume	476.72	4.06	0.04	0.01
	Colony size + Nesting‐support quality + Nest volume	476.77	4.11	0.04	0.02
	Nesting‐support quality + Laying period + Nest volume	477.15	4.49	0.03	0.02
	Colony size	478.17	5.51	0.02	0.00
	Year + Nest volume	478.72	6.06	0.01	0.02
	Laying period	478.91	6.25	0.01	0.00
	Year + Nesting‐support quality + Nest volume	479.01	6.35	0.01	0.03
	Colony size + Nesting‐support quality	479.08	6.42	0.01	0.01
	Nesting‐support quality + Laying period	479.22	6.56	0.01	0.01
	Nest volume + Colony identity	479.54	6.88	0.01	0.03
	Colony size + Laying period + Nest volume	480.05	7.39	0.01	0.02
	Colony size + Nesting‐support quality + Laying period + Nest volume	480.97	8.31	0.00	0.02
	Year	480.99	8.33	0.00	0.00
	Nesting‐support quality + Nest volume + Colony identity	481.21	8.55	0.00	0.03
	Year + Nesting‐support quality	481.31	8.65	0.00	0.01
	Colony identity	481.91	9.25	0.00	0.01
	Year + Colony size + Nest volume	481.91	9.25	0.00	0.02
	Colony size + Laying period	482.30	9.64	0.00	0.00
	Year + Laying period + Nest volume	482.36	9.70	0.00	0.02
	Laying period + Nest volume + Colony identity	482.60	9.94	0.00	0.03
	Year + Colony size + Nesting‐support quality + Nest volume	482.72	10.06	0.00	0.03
	Year + Nesting‐support quality + Laying period + Nest volume	482.81	10.15	0.00	0.03
	Colony size + Nesting‐support quality + Laying period	483.05	10.39	0.00	0.01
	Nesting‐support quality + Colony identity	483.50	10.84	0.00	0.02
	Year + Colony size	483.99	11.33	0.00	0.01
	Year + Laying period	484.22	11.56	0.00	0.01
	Nesting‐support quality + Laying period + Nest volume + Colony identity	484.70	12.04	0.00	0.04
	Year + Nesting‐support quality + Laying period	484.81	12.15	0.00	0.02
	Year + Colony size + Nesting‐support quality	484.86	12.20	0.00	0.02
	Laying period + Colony identity	485.45	12.79	0.00	0.01
	Year + Nest volume + Colony identity	485.60	12.94	0.00	0.03
	Year + Colony size + Laying period + Nest volume	486.07	13.41	0.00	0.02
	Year + Colony size + Nesting‐support quality + Laying period + Nest volume	486.92	14.26	0.00	0.03
	Nesting‐support quality + Laying period + Colony identity	487.35	14.69	0.00	0.02
	Year + Colony identity	487.67	15.01	0.00	0.02
	Year + Nesting‐support quality + Nest volume + Colony identity	487.87	15.21	0.00	0.04
	Year + Colony size + Laying period	488.01	15.35	0.00	0.01
	Year + Colony size + Nesting‐support quality + Laying period	488.95	16.29	0.00	0.02
	Year + Laying period + Nest volume + Colony identity	489.44	16.78	0.00	0.03
	Year + Nesting‐support quality + Colony identity	489.91	17.25	0.00	0.02
	Year + Nesting‐support quality + Laying period + Nest volume + Colony identity	491.55	18.89	0.00	0.04
	Year + Laying period + Colony identity	491.94	19.28	0.00	0.02
	Year + Nesting‐support quality + Laying period + Colony identity	494.13	21.47	0.00	0.02
Mean egg volume	Nest volume + Colony identity	657.03	0.00	0.15	0.09
	Nest volume + Colony identity + Nesting‐support quality	657.29	0.26	0.13	0.10
	Nest volume + Colony identity + Laying period	657.43	0.40	0.12	0.10
	Nest volume + Colony identity + Nesting‐support quality + Laying period	658.13	1.09	0.09	0.10
	Nest volume + Colony identity + Year	658.60	1.57	0.07	0.10
	Year + Nesting‐support quality + Nest volume + Colony identity	659.07	2.04	0.05	0.11
	Laying period + Colony identity	659.40	2.37	0.04	0.08
	Colony identity	659.62	2.58	0.04	0.07
	Year + Laying period + Nest volume + Colony identity	659.67	2.64	0.04	0.10
	Year + Nesting‐support quality + Laying period + Nest volume + Colony identity	659.92	2.89	0.03	0.11
	Nesting‐support quality + Colony identity	659.93	2.90	0.03	0.08
	Nesting‐support quality + Laying period + Colony identity	660.20	3.17	0.03	0.09
	Nest volume + Clutch size + Colony identity	660.89	3.86	0.02	0.09
	Nesting‐support quality + Nest volume + Clutch size + Colony identity	660.97	3.94	0.02	0.10
	Laying period + Nest volume + Clutch size + Colony identity	661.19	4.15	0.02	0.10
	Year + Colony identity	661.24	4.21	0.02	0.08
	Year + Laying period + Colony identity	661.56	4.53	0.01	0.09
	Year + Nesting‐support quality + Colony identity	661.71	4.68	0.01	0.09
	Nesting‐support quality + Laying period + Nest volume + Clutch size + Colony identity	661.72	4.68	0.01	0.10
	Year + Nesting‐support quality + Laying period + Colony identity	661.75	4.71	0.01	0.10
	Year + Nest volume + Clutch size + Colony identity	662.47	5.43	0.01	0.10
	Laying period + Clutch size + Colony identity	662.55	5.51	0.01	0.09
	Year + Nesting‐support quality + Nest volume + Clutch size + Colony identity	662.81	5.78	0.01	0.11
	Clutch size + Colony identity	662.91	5.87	0.01	0.07
	Nesting‐support quality + Clutch size + Colony identity	662.92	5.89	0.01	0.08
	Nesting‐support quality + Laying period + Clutch size + Colony identity	663.09	6.05	0.01	0.09
	Year + Laying period + Nest volume + Clutch size + Colony identity	663.52	6.49	0.01	0.11
	Year + Nesting‐support quality + Laying period + Nest volume + Clutch size + Colony identity	663.61	6.57	0.01	0.11
	Year + Clutch size + Colony identity	664.55	7.52	0.00	0.09
	Year + Nesting‐support quality + Laying period + Clutch size + Colony identity	664.76	7.73	0.00	0.10
	Year + Nesting‐support quality + Clutch size + Colony identity	664.80	7.77	0.00	0.09
	Year + Laying period + Clutch size + Colony identity	664.85	7.81	0.00	0.09
	Nest volume	668.07	11.04	0.00	0.02
	Year + Nest volume	668.78	11.75	0.00	0.03
	Laying period + Nest volume	669.42	12.38	0.00	0.02
	Nesting‐support quality + Nest volume	670.00	12.97	0.00	0.02
	Colony size + Nest volume	670.12	13.09	0.00	0.02
	*null*	670.37	13.33	0.00	0.00
	Year + Laying period + Nest volume	670.80	13.77	0.00	0.03
	Year + Colony size + Nest volume	670.83	13.80	0.00	0.03
	Year + Nesting‐support quality + Nest volume	670.87	13.83	0.00	0.03
	Colony size + Laying period + Nest volume	671.04	14.00	0.00	0.02
	Year	671.20	14.17	0.00	0.01
	Nest volume + Clutch size	671.35	14.32	0.00	0.02
	Laying period	671.42	14.39	0.00	0.00
	Nesting‐support quality + Laying period + Nest volume	671.45	14.42	0.00	0.02
	Colony size + Nesting‐support quality + Nest volume	672.08	15.05	0.00	0.02
	Year + Nest volume + Clutch size	672.15	15.12	0.00	0.04
	Nesting‐support quality	672.29	15.26	0.00	0.00
	Colony size	672.41	15.38	0.00	0.00
	Year + Colony size + Laying period + Nest volume	672.73	15.70	0.00	0.03
	Laying period + Nest volume + Clutch size	672.78	15.74	0.00	0.03
	Clutch size	672.89	15.85	0.00	0.01
	Year + Nesting‐support quality + Laying period + Nest volume	672.90	15.86	0.00	0.03
	Year + Colony size + Nesting‐support quality + Nest volume	672.95	15.92	0.00	0.03
	Colony size + Laying period	672.96	15.93	0.00	0.01
	Year + Laying period	673.00	15.97	0.00	0.02
	Colony size + Nesting‐support quality + Laying period + Nest volume	673.13	16.10	0.00	0.02
	Nesting‐support quality + Nest volume + Clutch size	673.22	16.18	0.00	0.02
	Year + Colony size	673.26	16.22	0.00	0.01
	Year + Nesting‐support quality	673.26	16.23	0.00	0.01
	Colony size + Nest volume + Clutch size	673.43	16.39	0.00	0.02
	Nesting‐support quality + Laying period	673.46	16.43	0.00	0.01
	Year + Clutch size	673.81	16.78	0.00	0.02
	Laying period + Clutch size	674.07	17.03	0.00	0.01
	Year + Colony size + Nest volume + Clutch size	674.21	17.17	0.00	0.04
	Year + Nesting‐support quality + Nest volume + Clutch size	674.22	17.19	0.00	0.04
	Year + Laying period + Nest volume + Clutch size	674.23	17.20	0.00	0.04
	Colony size + Laying period + Nest volume + Clutch size	674.35	17.32	0.00	0.03
	Colony size + Nesting‐support quality	674.36	17.32	0.00	0.00
	Nesting‐support quality + Clutch size	674.71	17.67	0.00	0.01
	Nesting‐support quality + Laying period + Nest volume + Clutch size	674.78	17.74	0.00	0.03
	Year + Colony size + Laying period	674.83	17.80	0.00	0.02
	Year + Colony size + Nesting‐support quality + Laying period + Nest volume	674.86	17.83	0.00	0.04
	Colony size + Clutch size	674.95	17.91	0.00	0.01
	Colony size + Nesting‐support quality + Laying period	675.05	18.01	0.00	0.01
	Year + Nesting‐support quality + Laying period	675.07	18.03	0.00	0.02
	Colony size + Nesting‐support quality + Nest volume + Clutch size	675.32	18.28	0.00	0.02
	Year + Colony size + Nesting‐support quality	675.35	18.32	0.00	0.01
	Colony size + Laying period + Clutch size	675.54	18.50	0.00	0.01
	Year + Laying period + Clutch size	675.72	18.69	0.00	0.02
	Year + Nesting‐support quality + Clutch size	675.84	18.80	0.00	0.02
	Year + Colony size + Clutch size	675.87	18.83	0.00	0.02
	Nesting‐support quality + Laying period + Clutch size	676.05	19.01	0.00	0.01
	Year + Colony size + Laying period + Nest volume + Clutch size	676.15	19.11	0.00	0.04
	Year + Nesting‐support quality + Laying period + Nest volume + Clutch size	676.31	19.27	0.00	0.04
	Year + Colony size + Nesting‐support quality + Nest volume + Clutch size	676.32	19.29	0.00	0.04
	Colony size + Nesting‐support quality + Laying period + Nest volume + Clutch size	676.44	19.41	0.00	0.03
	Colony size + Nesting‐support quality + Clutch size	676.79	19.76	0.00	0.01
	Year + Colony size + Nesting‐support quality + Laying period	676.94	19.91	0.00	0.02
	Year + Colony size + Laying period + Clutch size	677.52	20.49	0.00	0.02
	Colony size + Nesting‐support quality + Laying period + Clutch size	677.61	20.57	0.00	0.01
	Year + Nesting‐support quality + Laying period + Clutch size	677.73	20.70	0.00	0.02
	Year + Colony size + Nesting‐support quality + Clutch size	677.93	20.90	0.00	0.02
	Year + Colony size + Nesting‐support quality + Laying period + Nest volume + Clutch size	678.27	21.24	0.00	0.04
	Year + Colony size + Nesting‐support quality + Laying period + Clutch size	679.60	22.56	0.00	0.02

## References

[ece38162-bib-0001] Adam, G. , & Elie, P. (1993). Etude de la faune ichthyologique et de l'exploitation halieutique professionnelle du Lac de Grand Lieu, Loire Atlantique. CEMAGREF, MNHN.

[ece38162-bib-0002] Arnold, T. W. (2010). Uninformative parameters and model selection using Aikake's information criterion. Journal of Wildlife Management, 74, 1175–1178. 10.1111/j.1937-2817.2010.tb01236.x

[ece38162-bib-0003] Bakaria, F. , Rizi, H. , Ziane, N. , Chabi, Y. , & Banbura, J. (2002). Breeding ecology of whiskered terns in Algeria, North Africa. Waterbirds, 25, 56–62. 10.1675/1524-4695(2002)025[0056:BEOWTI]2.0.CO;2

[ece38162-bib-0004] Birchard, G. F. , & Deeming, D. C. (2015). Egg allometry: Influences of phylogeny and the altricial‐precocial continuum. In D. C. Deeming & S. J. Reynolds (Eds.), Nests, eggs, and incubation: New ideas about avian reproduction (pp. 97–112). Oxford University Press.

[ece38162-bib-0005] Britt, J. , & Deeming, D. C. (2011). First egg date and air temperature affect nest construction in blue tits *Cyanistes caeruleus* but not in great tits *Parus major* . Bird Study, 58, 78–89. 10.1080/00063657.2010.524916

[ece38162-bib-0006] Brown, C. R. (2016). The ecology and evolution of colony‐size variation. Behavioral Ecology and Sociobiology, 70, 1613–1632. 10.1007/s00265-016-2196-x

[ece38162-bib-0007] Brulez, K. , Pike, T. W. , & Reynolds, S. J. (2015). Egg signaling: The use of visual, auditory, and chemical stimuli. In D. C. Deeming & S. J. Reynolds (Eds.), Nests, eggs, and incubation: New ideas about avian reproduction (pp. 127–141). Oxford University Press.

[ece38162-bib-0008] Burley, N. (1986). Sexual selection for aesthetic traits in species with biparental care. American Naturalist, 127, 415–445. 10.1086/284493

[ece38162-bib-0009] Burnham, K. P. , & Anderson, D. R. (2003). Model selection and multimodel inference: A practical information‐theoretic approach. Springer Science & Business Media.

[ece38162-bib-0010] Carrascal, L. M. , Moreno, J. , & Amat, J. A. (1995). Nest maintenance and stone theft in the chinstrap penguin (*Pygoscelis antarctica*). 2. Effects of breeding group size. Polar Biology, 15, 541–545. 10.1007/BF00239645

[ece38162-bib-0011] Chambon, R. , Latraube, F. , Bretagnolle, V. , & Paillisson, J. M. (2020). Sex‐specific contributions to reproduction in whiskered tern *Chlidonias hybrida* colonies of varying breeding density. Ardeola, 67, 113–125. 10.13157/arla.67.1.2020.sc6

[ece38162-bib-0012] Collias, N. E. , & Collias, E. C. (1984). Nest building and bird behavior. Princeton University Press.

[ece38162-bib-0013] Coulson, J. C. (1963). Egg size and shape in the kittiwake (*Rissa tridactyla*) and their use in estimating age composition of populations. Proceedings of the Zoological Society of London, 140, 211–227. 10.1111/j.1469-7998.1963.tb01861.x

[ece38162-bib-0014] Coulson, J. C. , & Porter, J. M. (1985). Reproductive success of the kittiwake *Rissa tridactyla*: The roles of clutch size, chick growth rates and parental quality. Ibis, 127, 450–466. 10.1111/j.1474-919X.1985.tb04841.x

[ece38162-bib-0015] Coulson, J. C. , & Thomas, C. (1985). Difference in the breeding performance of individual kittiwake gulls, *Rissa tridactyla* (L.). In R. M. Sibly & R. H. Smith (Eds.), Behavioural ecology: Ecological consequences of adaptive behaviour (pp. 489–503). Blackwell.

[ece38162-bib-0016] Deeming, D. C. , & Mainwaring, M. C. (2015). Functional properties of nests. In D. C. Deeming & S. J. Reynolds (Eds.), Nests, eggs, and incubation: New ideas about avian reproduction (pp. 29–49). Oxford University Press.

[ece38162-bib-0017] Gochfeld, M. , & Burger, J. (1996). Family Sternidae (terns). In J. del Hoyo , A. Eliott & J. Sargatal (Eds.), Handbook of the birds of the World. Hoatzin to auks (Vol. 3 pp. 624–667). Lynx edicions.

[ece38162-bib-0018] Gustafsson, L. , Nordling, M. D. , Anderson, M. S. , Sheldon, B. C. , & Qvarnström, A. (1994). Infectious diseases, reproductive effort and the cost of reproduction in birds. Philosophical Transactions of the Royal Society B: Biological Sciences, 346, 323–331. 10.1098/rstb.1994.0149 7708827

[ece38162-bib-0019] Hansell, M. H. (2000). Bird nests and construction behaviour. Cambridge University Press.

[ece38162-bib-0020] Hipfner, J. M. , Gaston, A. J. , Martin, D. L. , & Jones, I. L. (1999). Seasonal declines in replacement egg‐layings in a long‐ lived, Arctic seabird: Costs of late breeding or variation in female quality? Journal of Animal Ecology, 68, 988–998. 10.1046/j.1365-2656.1999.00346.x

[ece38162-bib-0021] Hoelzer, G. A. (1989). The good parent process of sexual selection. Animal Behavior, 38, 1067–1078. 10.1016/S0003-3472(89)80146-0

[ece38162-bib-0022] Hoi, H. , Schleicher, B. , & Valera, F. (1994). Female mate choice and nest desertion in penduline tits, *Remiz pendulinus*: The importance of nest quality. Animal Behavior, 48, 743–746. 10.1006/anbe.1994.1296

[ece38162-bib-0023] Ketterson, E. D. , & Nolan, V. Jr. (1983). The evolution of differential bird migration. In R. F. Johnston (Ed.), Current ornithology (Vol. 1, pp. 357–402). Plenum Press.

[ece38162-bib-0024] Latraube, F. (2006). Biologie de la reproduction de la Guifette moustac Chlidonias hybrida en Brenne. Mémoire de l'Ecole Pratique des Hautes Etudes. Université de Montpellier II.

[ece38162-bib-0025] Ledwoń, M. (2011). Sexual size dimorphism, assortative mating and sex identification in the Whiskered Tern *Chlidonias hybrida* . Ardea, 99, 191–198. 10.5253/078.099.0209

[ece38162-bib-0026] Ledwoń, M. , & Neubauer, G. (2017). Offspring desertion and parental care in the whiskered tern *Chlidonias hybrida* . Ibis, 159, 860–872. 10.1111/ibi.12496 34800441

[ece38162-bib-0027] Liker, A. , & Székely, T. (2005). Mortality costs of sexual selection and parental care in natural populations of birds. Evolution, 59, 890–897. 10.1111/j.0014-3820.2005.tb01762.x 15926698

[ece38162-bib-0028] Mainwaring, M. C. , & Hartley, I. R. (2008). Seasonal adjustments in nest cup lining in blue tits *Cyanistes caeruleus* . Ardea, 96, 278–282. 10.5253/078.096.0213

[ece38162-bib-0029] Mainwaring, M. C. , & Hartley, I. R. (2013). The energetic costs of nest building in birds. Avian Biology Research, 6, 12–17. 10.3184/175815512X13528994072997

[ece38162-bib-0030] Mainwaring, M. C. , Reynolds, S. J. , & Weidinger, K. (2015). The influence of predation on the location and design of nests. In D. C. Deeming & S. J. Reynolds (Eds.), Nests, eggs, and incubation: New ideas about avian reproduction (pp. 50–64). Oxford University Press.

[ece38162-bib-0031] Minias, P. , Minias, A. , & Dziadek, J. (2014). Occurrence of extra‐pair paternity and intraspecific brood parasitism in the whiskered tern *Chlidonias hybrida* . Bird Study, 61, 130–134. 10.1080/00063657.2013.860949

[ece38162-bib-0032] Møller, A. P. (1994). Sexual selection and the barn swallow. Oxford University Press.

[ece38162-bib-0033] Moore, D. J. , Williams, T. D. , & Morris, R. D. (2000). Mate provisioning, nutritional requirements for egg production, and primary reproductive effort of female common terns *Sterna hirundo* . Journal of Avian Biology, 31, 183–196. 10.1034/j.1600-048X.2000.310210.x

[ece38162-bib-0034] Moreno, J. , Bustamante, J. , & Viñuela, J. (1995). Nest maintenance and stone theft in the chinstrap penguin (*Pygoscelis antarctica*): 1. Sex roles and effects on fitness. Polar Biology, 15, 533–540. 10.1007/BF00239644

[ece38162-bib-0035] Moreno, J. , Soler, M. , Møller, A. P. , & Linden, M. (1994). The function of stone carrying in the black wheatear, *Oenanthe leucura* . Animal Behavior, 47, 1297–1309. 10.1006/anbe.1994.1178

[ece38162-bib-0036] Muth, F. , & Healy, S. D. (2011). The role of adult experience in nest building in the zebra finch, *Taeniopygia guttata* . Animal Behaviour, 82, 185–189. 10.1016/j.anbehav.2011.04.021

[ece38162-bib-0037] Mužinić, J. , & Delić, A. (1997). Nesting biology of whiskered tern *Chlidonias hybridus* in Croatia. Avocetta, 21, 165–168.

[ece38162-bib-0038] Nagy, J. , Hauber, M. E. , Hartley, I. R. , & Mainwaring, M. C. (2019). Correlated evolution of nest and egg characteristics in birds. Animal Behaviour, 158, 211–215. 10.1016/j.anbehav.2019.10.015

[ece38162-bib-0039] Neuman, J. , Chardine, J. W. , & Porter, J. M. (1998). Courtship feeding and reproductive success in black‐legged kittiwakes. Colonial Waterbirds, 21, 73–80.

[ece38162-bib-0040] Nisbet, I. (1973). Courtship‐feeding, egg‐size and breeding success in common terns. Nature, 241, 141–142. 10.1038/241141a0

[ece38162-bib-0041] Paillisson, J. M. , Latraube, F. , Marion, L. , & Bretagnolle, V. (2008). Indirect evidence of conspecific nest parasitism in the colonial whiskered tern *Chlidonias hybrida* . Comptes Rendus Biologies, 331, 559–567. 10.1016/j.crvi.2008.04.010 18558379

[ece38162-bib-0042] Paillisson, J. M. , & Marion, L. (2006). Can small water level fluctuations affect the biomass of *Nymphaea alba* in large lakes? Aquatic Botany, 84, 259–266. 10.1016/j.aquabot.2005.10.004

[ece38162-bib-0043] Paillisson, J. M. , & Marion, L. (2011). Water level fluctuations for managing excessive plant biomass in shallow lakes. Ecological Engineering, 37, 241–247. 10.1016/j.ecoleng.2010.11.017

[ece38162-bib-0044] Paillisson, J.‐M. , Reeber, S. , Carpentier, A. , & Marion, L. (2006). Plant‐water regime management in a wetland: Consequences for a floating vegetation‐nesting bird, whiskered tern *Chlidonias hybridus* . Biodiversity and Conservation, 15, 3469–3480. 10.1007/s10531-004-2939-2

[ece38162-bib-0045] Paillisson, J. M. , Reeber, S. , Carpentier, A. , & Marion, L. (2007). Reproductive parameters in relation to food supply in the whiskered tern (*Chlidonias hybrida*). Journal of Ornithology, 148, 69–77. 10.1007/s10336-006-0102-4

[ece38162-bib-0046] Palomino, J. J. , Martín‐Vivaldi, M. , Soler, M. , & Soler, J. J. (1998). Functional significance of nest size variation in the Rufous bush robin *Cercotrichas galactotes* . Ardea, 86, 177–185.

[ece38162-bib-0047] Parsons, J. (1975). Seasonal variation in the breeding success of the herring gull: An experimental approach to pre‐fledging success. Journal of Animal Ecology, 44, 553–573. 10.2307/3611

[ece38162-bib-0048] Persson, O. , & Öhrström, P. (1996). Female nest choice in the penduline tit: A comment on Hoi et al (1994). Animal Behaviour, 51(2), 462–463. 10.1006/anbe.1996.0045

[ece38162-bib-0049] R Development Core Team (2018). R: A language and environment for statistical computing. R Foundation for Statistical Computing. http://www.R‐project.org

[ece38162-bib-0050] Slagsvold, T. , & Lifjeld, J. T. (1990). Influence of male and female quality on clutch size in tits (*Parus* spp.). Ecology, 71, 1258–1266. 10.2307/1938263

[ece38162-bib-0051] Smith, J. A. , Harrison, T. J. E. , Martin, G. R. , & Reynolds, S. J. (2013). Feathering the nest: Food supplementation influences nest construction by Blue (*Cyanistes caeruleus*) and Great Tits (*Parus major*). Avian Biol Res, 6, 18–25. 10.3184/175815512X13530764553094

[ece38162-bib-0052] Soler, J. J. , De Neve, L. , Martínez, J. G. , & Soler, M. (2001). Nest size affects clutch size and the start of incubation in magpies: An experimental study. Behavioral Ecology, 12, 301–307. 10.1093/beheco/12.3.301

[ece38162-bib-0053] Soler, J. J. , Møller, A. P. , & Soler, M. (1998). Nest building, sexual selection and parental investment. Evolutionary Ecology, 12, 427–441. 10.1023/A:1006520821219

[ece38162-bib-0054] Stearns, S. C. (1992). The evolution of life histories. Oxford University Press.

[ece38162-bib-0055] Sydeman, W. J. , Penniman, J. F. , Penniman, T. M. , Pyle, P. , & Ainley, D. G. (1991). Breeding performance in the western gull: Effects of parental age, timing of breeding and year in relation to food availability. Journal of Animal Ecology, 60, 135–149. 10.2307/5450

[ece38162-bib-0056] Van der Winden, J. , Beintema, A. J. , & Heemskerk, L. (2004). Habitat‐related black tern *Chlidonias niger* breeding success in The Netherlands. Ardea, 92, 53–61.

[ece38162-bib-0057] Verhulst, S. , & Nilsson, J. Å. (2008). The timing of birds' breeding seasons: A review of experiments that manipulated timing of breeding. Philosophical Transactions of the Royal Society B: Biological Sciences, 363, 399–410. 10.1098/rstb.2007.2146 PMC2606757 17666390

[ece38162-bib-0058] Verhulst, S. , van Balen, J. H. , & Tinbergen, J. M. (1995). Seasonal decline in reproductive success of the great tit: Variation in time or quality? Ecology, 76, 2392–2403. 10.2307/2265815

[ece38162-bib-0059] Wachtmeister, C. A. (2001). Display in monogamous pairs: A review of empirical data and evolutionary explanations. Animal Behavior, 61, 861–868. 10.1006/anbe.2001.1684

[ece38162-bib-0060] Wendeln, H. (1997). Body mass of female common terns (*Sterna hirundo*) during courtship: Relationships to male quality, egg mass, diet, laying date and age. Colon Waterbirds, 20, 235–243. 10.2307/1521689

[ece38162-bib-0061] Wiggins, D. A. , & Morris, R. D. (1986). Criteria for female choice of mates: Courtship feeding and parental care in the common tern. American Naturalist, 128, 126–129. 10.1086/284545

[ece38162-bib-0062] Williams, J. B. (1986). Energetics of avian incubation. In C. Carey (Ed.), Avian energetics and nutritional ecology (pp. 375–415). Chapman & Hall.

[ece38162-bib-0063] Yoon, J. , Ha, H. S. , Jung, J. S. , & Park, S. R. (2015). Post‐mating sexual behaviors of oriental storks (*Ciconia boyciana*) in Captivity. Zoological Science, 32, 331–335. 10.2108/zs140178 26245219

